# Effect of multiple micronutrient supplements *v*. iron and folic acid supplements on neonatal mortality: a reanalysis by iron dose

**DOI:** 10.1017/S1368980022001008

**Published:** 2022-08

**Authors:** Filomena Gomes, Rina Agustina, Robert E Black, Parul Christian, Kathryn G Dewey, Klaus Kraemer, Anuraj H Shankar, Emily Smith, Alison Tumilowicz, Megan W Bourassa

**Affiliations:** 1 The New York Academy of Sciences, New York, NY 10006, USA; 2 NOVA Medical School, Lisbon, Portugal; 3 Department of Nutrition, Faculty of Medicine, Universitas Indonesia – Dr Cipto Mangunkusumo General Hospital, Jakarta, Indonesia; 4 Human Nutrition Research Centre, Indonesian Medical Education and Research Institute, Faculty of Medicine, Universitas Indonesia, Jakarta, Indonesia; 5 Johns Hopkins Bloomberg School of Public Health, Baltimore, MD, USA; 6 University of California, Davis, Davis, CA, USA; 7 Sight and Life Foundation, Basel, Switzerland; 8 University of Oxford, Oxford, UK; 9 Summit Institute for Development, Mataram, Indonesia; 10 The George Washington University, Washington, DC, USA; 11 The Bill & Melinda Gates Foundation, Seattle, Washington, DC, USA

**Keywords:** Micronutrient supplements, Iron, Pregnancy, Neonatal mortality

## Abstract

**Objective:**

Antenatal multiple micronutrient supplements (MMS) are a cost-effective intervention to reduce adverse pregnancy and birth outcomes. However, the current WHO recommendation on the use of antenatal MMS is conditional, partly due to concerns about the effect on neonatal mortality in a subgroup of studies comparing MMS with iron and folic acid (IFA) supplements containing 60 mg of Fe. We aimed to assess the effect of MMS *v*. IFA on neonatal mortality stratified by Fe dose in each supplement.

**Methods:**

We updated the neonatal mortality analysis of the 2020 WHO guidelines using the generic inverse variance method and applied the random effects model to calculate the effect estimates of MMS *v*. IFA on neonatal mortality in subgroups of trials (*n* 13) providing the same or different amounts of Fe, that is, MMS with 60 mg of Fe *v*. IFA with 60 mg of Fe; MMS with 30 mg of Fe *v*. IFA with 30 mg of Fe; MMS with 30 mg of Fe *v*. IFA with 60 mg of Fe; and MMS with 20 mg of Fe *v*. IFA with 60 mg of Fe.

**Results:**

There were no statistically significant differences in neonatal mortality between MMS and IFA within any of the subgroups of trials. Analysis of MMS with 30 mg *v*. IFA with 60 mg of Fe (7 trials, 14 114 participants), yielded a non-significant risk ratio of 1·12 (95 % CI 0·83 to 1·50).

**Conclusion:**

Neonatal mortality did not differ between MMS and IFA regardless of Fe dose in either supplement.

## Overview of WHO analyses on neonatal mortality

The neonatal period (the first 28 d of life) is the most vulnerable time for child survival. Although the global number of neonatal deaths has declined from 5 million in 1990 to 2·4 million in 2019, they now comprise 47 % of all child deaths under the age of 5 years. The most common causes of neonatal mortality (death in the first 28 d following a live birth) are preterm birth, intrapartum complications leading to birth asphyxia, infections and congenital defects^(1)^. In 2019, sub-Saharan Africa had the highest neonatal mortality rate (27 deaths/1000 live births), followed by Central and Southern Asia (24 deaths/1000 live births)^(1)^.

Prenatal multiple micronutrient supplements (MMS) containing iron and folic acid (IFA) are a cost-effective intervention to reduce adverse pregnancy and birth outcomes^(2–5)^. Growing evidence in favour of MMS led to an update in the global recommendation of prenatal MMS, such that the most recent 2020 WHO guidelines^(6)^ now recommend this intervention ‘in the context of rigorous research’. This remains a conditional recommendation in part because the guideline development group expressed concerns based on a sensitivity analysis in a subgroup of trials possibly showing a higher risk of neonatal mortality with MMS *v*. IFA providing 60 mg of Fe.

The studies included for analysis in the 2020 WHO guidelines^(6)^ compared the effect of MMS *v*. IFA on a number of outcomes and provided a range of Fe dose, from 20 mg to 60 mg of Fe in MMS and either 30 mg or 60 mg of Fe in IFA. Most MMS, including the widely used and well-established UNICEF/WHO/United Nations University International Multiple Micronutrient Preparation (UNIMMAP) formulation, contain 30 mg of elemental Fe^(7)^, consistent with the WHO recommended 30–60 mg of Fe for IFA^(8)^. The neonatal mortality analyses in the WHO guidelines^(6)^ included two comparisons: (1) MMS (any Fe dose, from 20 mg to 60 mg) *v*. IFA (30 mg or 60 mg of Fe) and (2) MMS UNIMMAP (30 mg of Fe) *v*. IFA (30 mg or 60 mg of Fe). A subsequent sensitivity analysis was limited to the trials that provided 400 mg of folic acid in the IFA group, excluding two studies that used a higher or lower amount of folic acid in the IFA group. The results of the neonatal mortality analyses of the 2020 WHO guidelines are summarised in Table 1. In both comparisons 1 and 2, results for the subgroup of MMS *v*. IFA with 60 mg of Fe suggested that MMS was associated with an increased risk of neonatal mortality when the analyses were limited to the trials using 400 mg of folic acid. Based on these results, the 2020 WHO guidelines^(6)^ concluded that ‘when compared with IFA supplements containing a higher dose of Fe (60 mg), MMS may be less effective in reducing neonatal mortality’.

**Table 1:** Summary of 2020 WHO guidelines^(6)^ comparing the effect of MMS *v*. IFA on neonatal mortality, both overall and sensitivity analysis

Subgroup	Overall analyses				Sensitivity analysis limited to trials with 0·4 mg of folic acid		
	Number of studies (studies)	Risk ratio for neonatal mortality (95 % CI)		*P*-value for subgroup differences	Number of studies (studies)	Risk ratio for neonatal mortality	95 % CI
Comparison 1				0·08			
MMS (any Fe dose) *v*. IFA with 60 mg of Fe	9 (a, b, c, d, e, g, h, j, m)	1·22	0·94, 1·56		8 (a, b, c, d, e, g, h, m)	1·32	1·05, 1·65
MMS (any Fe dose) *v*. IFA with 30 mg of Fe	4 (f, i, k, l)	0·95	0·87, 1·04		3 (f, i, k)	Not reported	
Comparison 2				0·05			
MMS UNIMMAP (30 mg of Fe) *v*. IFA with 60 mg of Fe	6 (b, e, g, h, j, m)	1·25	0·94, 1·67		5 (b, e, g, h, m)	1·38	1·05, 1·82
MMS UNIMMAP (30 mg of Fe) *v*. IFA with 30 mg of Fe	3 (f, i, k)	0·90	0·78, 1·05		3 (f, i, k)	0·90	0·78, 1·05

MMS, multiple micronutrient supplements; IFA, iron and folic acid; a, Ashorn 2010; b, Bhuta 2009; c, Christian 2003; d, Dewey 2009; e, Kaestel 2005; f, Liu 2013; g, Osrin 2005; h, Roberfroid 2008; i, SUMMIT 2008; j, Sunawang 2009; k, Tofail 2008; l, West 2014; m, Zeng 2008.

### A reanalysis of the WHO neonatal mortality data

The MMS Technical Advisory Group^(9)^, hosted by New York Academy of Sciences, determined that a reanalysis of the neonatal mortality data from trials included in the 2020 WHO guidelines^(6)^ according to Fe dose provided by MMS and IFA would be useful. Given the wide range of Fe doses used in the studies included in those analyses^(6)^, we reanalysed the data presented in therein using four comparison groups according to Fe dose. Thus, we aimed to assess the effect of MMS *v*. IFA on neonatal mortality in the subgroups of studies providing MMS with 60 mg of Fe *v*. IFA with 60 mg of Fe, MMS with 30 mg of Fe *v*. IFA with 30 mg of Fe, MMS with 30 mg of Fe *v*. IFA with 60 mg of Fe and MMS with 20 mg of Fe *v*. IFA with 60 mg of Fe.

In our analyses, we identified and corrected the following methodological issues and made additional adjustments.

First, the 2020 WHO guidelines’^(6)^ sensitivity analyses were limited to trials that provided IFA with 400 mg of folic acid, which led to removal of the Sunawang 2009 trial^(10)^ wherein the control group received 250 mg of folic acid, and removal of the large West 2014 trial with 44 567 pregnant women^(11)^ wherein MMS and control group received 600 mg of folic acid rather than 400 mg. The WHO guideline development group’s rationale for excluding trials based on the folic acid dose was ‘if countries are to consider transitioning to MMS, they would most likely be switching from one of these two IFA formulations (i.e. 30 mg of Fe/400 mg of folic acid or 60 mg of Fe/400 mg of folic acid)’. However, this variation in the dose of folic acid is likely irrelevant to the Fe dose question. Folic acid supplementation has been shown to reduce neonatal mortality from neural tube disorders^(12)^, but women should take a folic acid supplement as early as possible, ideally before conception, to prevent neural tube defects^(8,13)^. Most of these trials had a mean gestational age at enrolment that falls into the second trimester of gestation; they rarely started at the beginning of the pregnancy, as recommended, when folic acid supplementation has an especially critical role. In addition, in the West trial, 0·6 mg of folic acid was provided in both groups and, as such, any difference between groups cannot be attributed to folic acid. Thus, we have not conducted sensitivity analyses based on dose of folic acid provided in the supplements.

Second, the Tofail 2008 trial^(14,15)^ included six arms (three receiving early food supplementation and three receiving usual food supplementation), which have been merged into three according to dose of Fe: MMS with 30 mg of Fe, IFA with 30 mg of Fe and IFA with 60 mg of Fe. The neonatal mortality analysis of the 2020 WHO guidelines^(6)^ excluded the group providing IFA with 60 mg, which we included in our reanalysis as it provides relevant data.

Third, the Kaestel 2005 trial^(16)^ also included three arms: MMS with 30 mg of Fe, MMS with 60 mg of Fe and IFA with 60 mg of Fe. The neonatal mortality analysis of the 2020 WHO guidelines^(6)^ generated, incorrectly, the same effect estimates for this trial in both comparisons (comparisons 1 and 2); however, comparison 2 should have been limited to the arm that provided 30 mg of Fe in MMS *v*. IFA with 60 mg of Fe. In addition, the sample sizes of each study arm reported in both forest plots (comparison 1 and 2) were related to the total number of women randomised to each arm. Specifically, the 2020 WHO guidelines used 708 women for the IFA arm, 695 women in the MMS 30-mg Fe arm and 697 women in the MMS 60-mg Fe arm^(6)^, rather than the number of live births for which they had data (i.e. 519, 525 and 542, respectively). In our reanalysis, we used the total number of events (i.e. neonatal mortality) within the sample of live births for each group.

Fourth, the West 2014 trial^(17)^ provided 27 mg of Fe in MMS and IFA to match the US Institute of Medicine RDA for Fe in pregnancy^(18)^ and was excluded from comparison 2 of the neonatal mortality analysis of the 2020 WHO guidelines^(6)^. The analysis of chemical composition of the MMS and IFA tablets showed that the percentage of proposed Fe in MMS (27 mg) varied from 105 % to 112 %, which is equivalent to 28·4 mg and 30·2 mg of Fe, respectively. In addition, the composition of MMS recommended by WHO/WFP/UNICEF for pregnant women in emergency settings has 27 mg of Fe^(19)^. In our reanalysis, we therefore considered this study as providing about 30 mg of Fe in each study arm, thereby including it in the subgroup providing IFA with 30 mg of Fe *v*. MMS with 30 mg of Fe.

After making the above changes, we used the same methods as the neonatal mortality analysis of the 2020 WHO guidelines^(6)^, using the log risk ratio, standard error and number of participants in each study arm, for all the thirteen studies included in comparison 1. Subgroup analyses were done based on the four comparison groups by Fe dose provided by MMS and IFA. We extracted new and corrected data for the Tofail 2008^(14,15)^ and Kaestel 2005 trials^(16)^, where each of the three study arms (for both studies) were allocated to the appropriate comparison group. Similar to the neonatal mortality analysis of the 2020 WHO guidelines^(6)^, we used the generic inverse variance method and applied the random effects model to calculate the effect estimates of MMS *v*. IFA. We did not conduct a non-inferiority analysis, as we have deliberately chosen to follow the same methodology used for the 2020 WHO guidelines, that is, subgroup analyses according to Fe dose provided by the supplements.

## Results

**Fig. 1:**
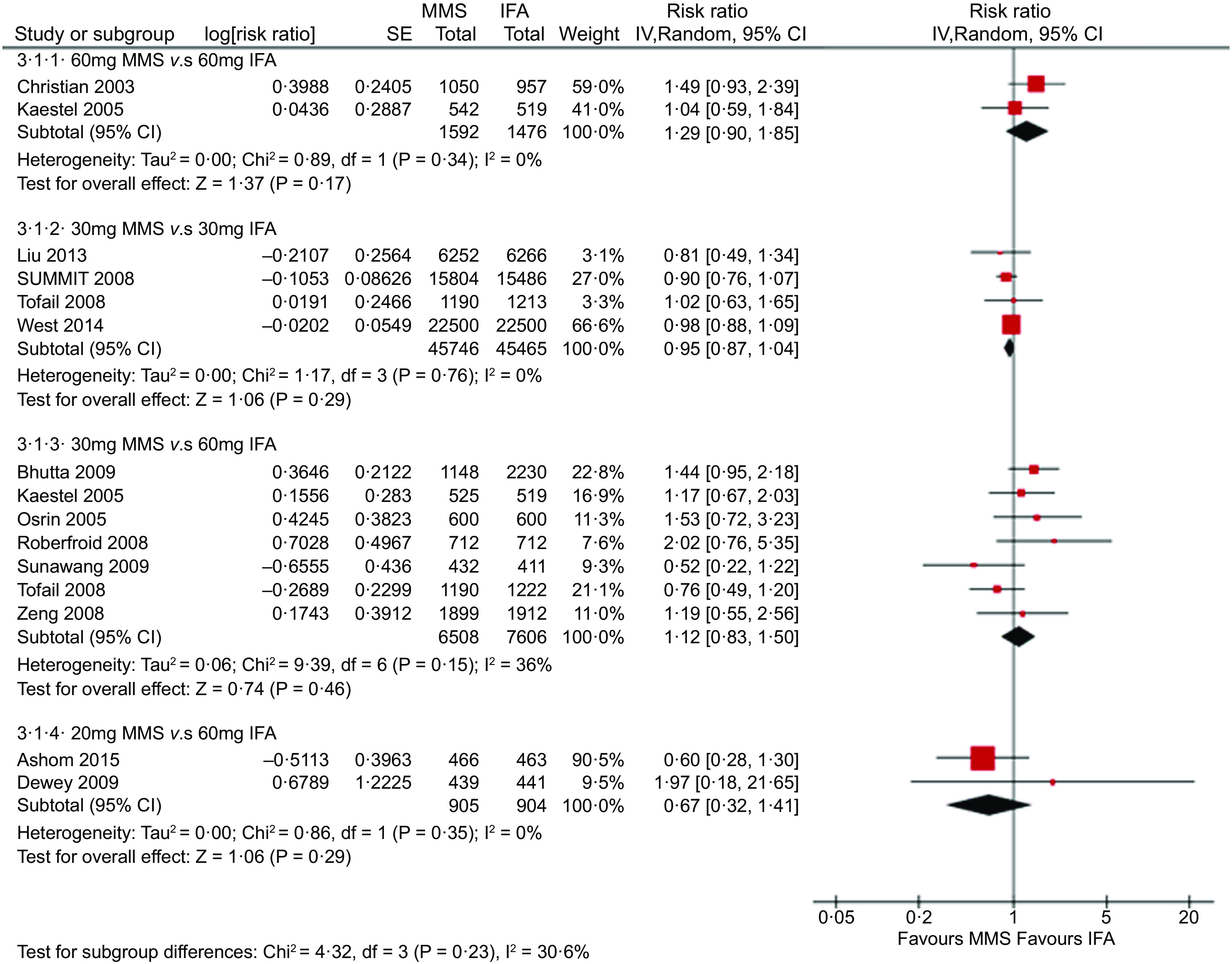
New analysis: effect of MMS *v*. IFA on neonatal mortality stratified by iron dose provided in each arm of thirteen trials. MMS, multiple micronutrient supplements; IFA, iron and folic acid

The forest plot of the effect of MMS *v*. IFA on neonatal mortality stratified by Fe dose (new analysis) is represented in Fig. 1. There were no statistically significant differences in neonatal mortality between MMS and IFA within any of the subgroups. This finding is consistent with the results of the previous Smith et al. 2017 two-stage meta-analysis of individual patient data and effect modifiers from seventeen randomised controlled trials from fourteen low-income and middle-income countries, which compared MMS and IFA in 112 953 pregnant women, and observed an overall neonatal mortality risk ratio of 0·99 (0·89–1·09)^(3)^.

Our main comparison of interest, which addresses the WHO concern regarding the effects of providing IFA with 60 mg of Fe *v*. MMS with 30 mg of Fe, included 7 studies with 14 114 participants and resulted in a risk ratio of 1·12 (95 % CI 0·83, 1·50) for neonatal mortality. While a possibility of increased risk cannot be excluded given this wide CI, MMS with 30 mg of Fe likely results in little or no difference in neonatal mortality, in comparison with IFA providing 60 mg of Fe.

In an additional analysis, we added the Fawzi 2007 trial^(20)^ (Appendix Figure 1), which was excluded from the WHO analyses because it did not meet the inclusion criterion of having at least thirteen micronutrients provided by MMS but was included in the 2017 individual patient data meta-analysis. The participants of this trial’s intervention group^(20)^ received MMS with vitamins B_1_, B_2_, B_3_, B_6_, B_9_, B_12_, C and E, in addition to Fe. The addition of this trial to the analyses did not change the conclusions. Specifically, no differences in neonatal mortality were observed between MMS and IFA in the subgroup of trials providing 60 mg of Fe in IFA and 60 mg of Fe in MMS.

Our results are also in line with a previous article that concluded there was no indication that MMS increases the risk of neonatal mortality in the subgroup of trials whose control groups provided 60 mg of Fe^(21)^. Furthermore, our results do not suggest that Fe dose plays an important role in this outcome; Fig. 1 shows that the comparison of 60 mg of Fe in MMS *v*. 60 mg of Fe in IFA tends to favour IFA, while the comparison of 20 mg of Fe in MMS *v*. 60 mg of Fe in IFA tends to favour MMS.

The removal of studies from the WHO analysis based on variations in folic acid dose, which likely have little clinical significance, led to a supposed increase in neonatal mortality with MMS in the WHO analysis. This could cause concern among countries that follow the WHO guidelines and, consequently, prevent the transition to MMS programmes and their associated well-documented benefits in reducing the risk of low birth weight, small for gestational age, stillbirth and preterm birth^(2,3)^.

## Conclusion

The present analysis of the neonatal mortality data from the thirteen trials included in the 2020 WHO guidelines^(6)^ suggests that this outcome does not differ between MMS and IFA regardless of Fe dose in either supplement – a finding consistent with other meta-analyses showing no risk, that is, neonatal mortality risk ratio of 0·99 (0·89–1·09)^(3)^. A transition from IFA containing 60 mg of Fe to MMS containing 30 mg of Fe would not adversely affect neonatal mortality. The WHO should consider the present analysis when updating the guidelines related to the use of MMS during pregnancy.

## Supplementary material

For supplementary material accompanying this paper visit https://doi.org/10.1017/S1368980022001008. click here to view supplementary material
