# Supplementary material for: Effect of multiple micronutrient supplements v. iron and folic acid supplements on neonatal mortality: a reanalysis by iron dose
Source: Public Health Nutr. 2022 Apr 25;25(8):2317–21. doi: 10.1017/S1368980022001008 (PMC9991737; doi:10.1017/S1368980022001008)

**Supplementary material**

Appendix Figure 1 - Effect of MMS vs IFA on neonatal mortality stratified by iron dose provided in each arm of 14 trials, including the Fawzi 2007 trial


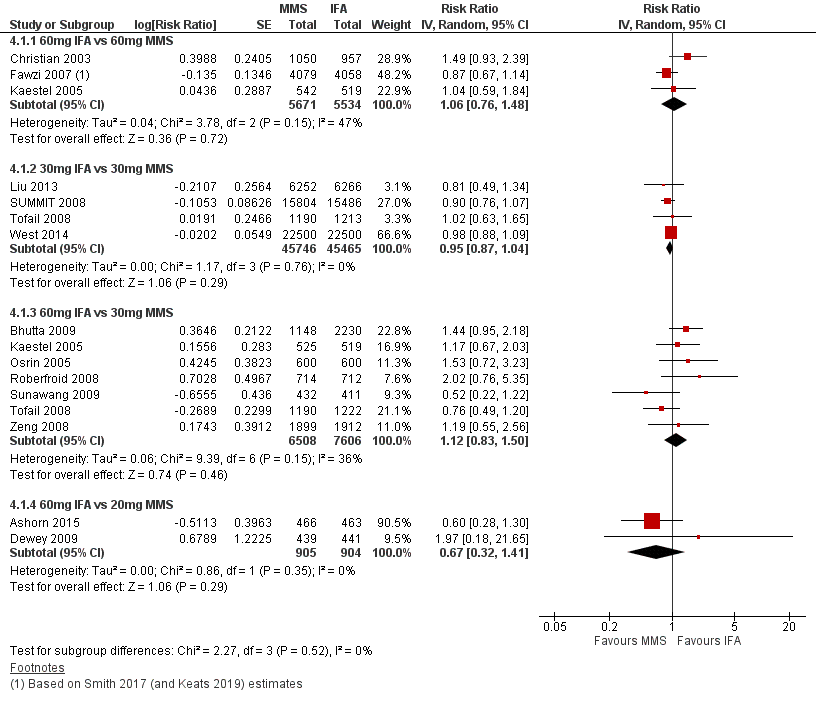

Supplement: Supplementary file 1 [file S1368980022001008sup001.docx]
